# Influence of Sanitizers on the Lipopolysaccharide Toxicity of *Escherichia coli* Strains Cultivated in the Presence of *Zygosaccharomyces bailii*

**DOI:** 10.1155/2014/135856

**Published:** 2014-05-25

**Authors:** Lerato Mogotsi, Olga De Smidt, Pierre Venter, Willem Groenewald

**Affiliations:** ^1^Unit for Applied Food Science and Biotechnology, Central University of Technology, Free State, P.O. Box 20539, Bloemfontein 9300, South Africa; ^2^Fonterra Co-Operative Group Limited, Private Bag 11029, Palmerston North 4442, Dairy Farm Road 1, Palmerston North, New Zealand

## Abstract

The influence of sublethal concentrations of two sanitizers, liquid iodophor and liquid hypochlorite (LH), on the growth rates and toxicity of food-borne pathogenic *Escherichia coli* strains grown in the presence of spoilage yeast *Zygosaccharomyces bailii* was assessed. When grown in combination with *Z. bailii* both *E. coli* O113 and *E. coli* O26 exhibited slower growth rates, except when *E. coli* O113 was grown in combination with *Z. bailii* at 0.2% LH. The growth rate of *Z. bailii* was not impacted by the addition of the sanitizers or by communal growth with *E. coli* strains. LAL and IL-6 results indicated a decrease in toxicity of pure *E. coli* cultures with comparable profiles for control and sanitizer exposed samples, although the LAL assay proved to be more sensitive. Interestingly, pure cultures of *Z. bailii* showed increased toxicity measured by LAL and decreased toxicity measured by IL-6. LAL analysis showed a decrease in toxicity of both *E. coli* strains grown in combination with *Z. bailii*, while IL-6 analysis of the mixed cultures showed an increase in toxicity. The use of LAL for toxicity determination in a mixed culture overlooks the contribution made by spoilage yeast, thus demonstrating the importance of using the appropriate method for toxicity testing in mixed microbe environments.

## 1. Introduction 

Microorganisms associated with food spoilage and food-borne diseases pose a considerable threat to human welfare and economy, especially that of developing countries [1]. Food is a complex material, more than often nutrient rich, and can generally support a diverse number of microorganisms that interact with each other and that can attach to a variety of surfaces to form biofilm in the food processing environment [2, 3]. Microorganisms associated with food spoilage and food-borne diseases are problematic in various sectors of the food industry and support continuous investigation.* Escherichia coli*, a Gram-negative bacterium, can be associated with food-borne diseases such as hemorrhagic colitis [4]. The last few decades have seen an increase in awareness of yeasts as food spoilage agents [5] and* Zygosaccharomyces bailii* has been described as the most important of all food spoilage yeasts [6]. This yeast originates from fruit trees exude and results in the spoilage of sweetened wine during processing. In addition,* Z. bailii* may lead to explosion of canned food as a result of gas production due to vigorous alcoholic fermentation [7].

To control food spoilage organisms and food-borne pathogens food processors have relied on physical and chemical methods [2]. However, if cleaning chemicals are not properly rinsed from food processing equipment they may end up in the product in sublethal concentrations. This could influence the response of the microorganisms to residual chemicals. Previous studies focused on the effect of sanitation procedures and sublethal doses of preservatives on the toxicity of specific microorganism [8]; however, these were conducted on pure cultures. There exists a need to investigate this phenomenon in conditions that more accurately reflect the situation found in industries where microorganisms exist not as pure cultures, but in communities.

In the food processing environment, a parallel exits between the resistance of microorganism and the efficiency of sanitizers. Resistance to sanitizers and preservatives may be attributed to cellular barriers of a microorganism such as the lipopolysaccharide (LPS) layer in Gram-negative bacteria that limits diffusion of molecules into the cell [9] and exopolysaccharide (EPS) from yeast cells, viral pathogens, and fungal spores [10]. The LPS layer has been shown to interfere with many host mediation systems, leading to hypotensive shock, disseminated intravascular coagulation, and metabolic abnormalities [11, 12]. Exopolysaccharides produced by attached cells assist the colonization of other organisms to surfaces leading to biofilm formation which are usually highly resistant to antibiotics [13]. The food industry relies on viable counts for microbial testing which do not take into account toxicity contributed by the remaining debris (LPS) of dead cells or the effect that membrane adaptation could have on toxicity of living cells. The limulus amoebocyte lysate (LAL), commonly used for endotoxin determination, can detect 3 pg mL^−1^ (0.03 EU mL^−1^) of LPS [14]. It does not detect other pyrogenic molecules, and numerous substances can interfere with the assay, for instance, the presence of inhibitory proteins in plasma [15]. The IL-6 ELISA method is used to determine the concentration of porcine specific IL-6 present within a sample. This assay can be performed on plasma, serum, and cell culture supernates. Thus, the aim of this study was to assess the effect of sublethal concentrations of sanitizers on communal growth of* E. coli *O113,* E. coli* O26, and* Z. bailii* and to monitor changes in toxicity as a result of sublethal concentrations of sanitizer exposure on pure and mixed cultures of* E. coli *and* Z. bailii* by means of LAL and IL-6 ELISA method.

## 2. Materials and Methods

### 2.1. Strains Used

*Escherichia coli* O113 (smooth strain) and* Escherichia coli* O26 (rough strain) were isolated from food samples and a spoilage yeast strain, while* Zygosaccharomyces bailii* Y-1535 was obtained from the University of Free State culture collection. The rough and smooth status of the* E. coli* were confirmed by the salt aggregation test method, which entailed the “salting out” of rough strains with 4 M ammonium sulphate [16].

### 2.2. Determining Sublethal Doses of Sanitizers

Liquid iodophor (LI) and liquid hypochlorite (LH) were purchased from a leading supplier of sanitizers for industrial use. A preliminary study was performed to determine sublethal concentrations by using the use-dilution method [17]. A series of dilutions of the sanitizers from 0.05%–0.6% LI and 0.075%–0.6% LH were inoculated with a loopful of either bacteria or yeast. The tubes were shaken at 200 rpm at 30°C and optical density was measured at different time intervals. Inadequate mixing and aeration in the tubes resulted in unusable data. Volumes were changed to 100 mL flasks and standardized inocula of initial OD_690nm_ values of 0.1 were used. Flasks were incubated at 30°C with shaking. Thus, the selected working concentrations in this study were as follows: 0.05% LH, 0.2% LH, and 0.075% LI. Two LH concentrations were used, in order to allow both* E. coli* strains to grow.* E*.* coli *O26 (rough strain) grew in the presence of 0.05% LH as it was more susceptible to 0.2% LH, while the growth of* E. coli* O113 (smooth) was less inhibited at this concentration.

### 2.3. Growth Conditions

At midexponential phase preinocula of* Escherichia coli* O113,* E. coli* O26 and* Z. bailii* Y-1535 were inoculated in 200 mL yeast malt (YM) media (Biolab, Midrand, South Africa) and incubated at 30°C with shaking at 200 rpm. Strains were cultivated as pure cultures and in combinations of* E. coli* and* Z. bailii*. Growth was monitored by optical density (OD_690 nm_) and viable plate counts on Violet Red Bile (Biolab) and YM media with 5% tartaric acid for* E. coli* and* Z. bailii,* respectively. Maximum specific growth rates (*μ*_ max _) were calculated by fitting a straight line to the data points that appeared to best represent the exponential growth phase using (1), where *N*_1_ and *N*_2_ are colony forming units at time 1 (*t*_1_) and time 2 (*t*_2_). Differences in growth of communal cultures from their corresponding control at two selected time intervals were calculated using (2) where [*T*_*C*_1__] represents concentration (%) of pure culture control (with sanitizer) at time 1 and [*T*_*G*_1__] represents the concentration (%) of communal growth (with sanitizer) at time 1. A value of zero was considered to indicate no difference in growth when the strains were grown in combination. When a difference between the two concentrations was below zero, the strain indicated better growth in combination and the opposite when the two concentrations were greater than zero:

(1) $$\begin{array}{c} \text{Ln}\frac{N_{2}/N_{1}}{t_{2}-t_{1}}, \end{array}$$

(2) $$\begin{array}{c} \left[T_{C_{1}}\right]-\left[T_{G_{1}}\right]=0. \end{array}$$

For toxicity analysis 1 mL of culture was sampled during the exponential growth phase (10 h* for E. coli* and 12 h for* Z. bailii*). The influence of the selected sanitizers was evaluated by growing the strains under the same conditions as described for the controls except for those with added sanitizers at predetermined concentrations of 0.2% or 0.05% liquid hypochlorite (LH) and 0.075% liquid iodophor (LI).

### 2.4. Limulus Amoebocyte Lysate Assay (LAL)

LPS toxicity was determined using the QCL-1000 chromogenic LAL assay (Lonza, Bloemfontein, South Africa) for all samples for time zero and at the late exponential phase of growth (10–12 h). This quantitative test for Gram-negative bacterial endotoxin was performed by the microplate method as prescribed by the manufacturer. The test kits included* E. coli* endotoxin standards with approximately 50–648 endotoxin units (EU) lyophilized endotoxins. Toxicity was calculated from a standard curve and change in toxicity was expressed as ΔEU · mL^−1^ · OD_690  nm^−1^_ which was converted to Δpg· mL^−1^ · OD_690  nm^−1^_ (1 EU = 100 pg endotoxin). Therefore, a positive value indicates an increase and a negative value a decrease in toxicity, while the Δtoxicity · OD_690  nm^−1^_ value represents the magnitude of change. The absorbance of released p-nitroaniline from the synthetic substrate resulting in a yellow colour was read at 410 nm with a microplate reader (Bio-Rad, Johannesburg, South Africa). All equipment used was pyrogen free.

### 2.5. Interleukin-6 (IL-6)

LPS/EPS toxicity on all samples from time zero and the late exponential phase of growth (10–12 h) was determined using the in vitro enzyme-linked immunosorbent assay for the quantitative measurement of porcine IL-6 (RayBiotech, Pretoria, South Africa). Blood was randomly collected from 10 pigs, pooled and diluted to 1 : 3 with sterile saline, and allowed to separate for 12 h at 37°C in a CO_2_ incubator. The supernatant was used for IL-6 determination measuring bound tetramethylbenzidine at 540 nm. The toxicity values were calculated from a standard curve. The change in toxicity values was expressed as Δpg· mL^−1^ · OD_690  nm^−1^_. Pyrogen free equipment, reagents, and consumables were used and all experiments were performed as independent duplicates.

## 3. Results and Discussion

### 3.1. Influence of Sanitizers on Maximum Specific Growth Rate of Pure and Mixed Cultures

The addition of sublethal concentrations of 0.075% LI resulted in no change in growth rates for* E. coli* O113 and O26 pure cultures compared to their unchallenged controls. However, the growth rate of mixed cultures of* E. coli* O113 and* Z. bailii and E. coli* O26 and* Z. bailii *showed a decrease with the addition of 0.075% LI as compared to the control which can be attributed to the influence of communal growth (Table 1).

Growth rate of* E. coli *O113 pure culture was noticeably impaired by exposure to 0.2% LH. However, the addition of a lower LH concentration (0.05%) did not influence the growth rate of* E. coli* O113 pure culture.

The growth rate of* E. coli *O26 pure culture was influenced by the addition of 0.05%. However, the addition of liquid iodophor resulted in no change in growth rate. Communal growth with* Z. bailii, *however, resulted in markedly slower growth rates. No negative impact was observed for* Z. bailii *growth rates by either communal growth with* E. coli *strains or the exposure to sublethal concentration of sanitizers. Interaction is considered to have taken place when the growth rate of the target microorganism in a mixed culture is decreased by 10% [11]. Generally, communal growth had a marked impact on the growth rates of the* E. coli* strains compared to their controls (pure cultures), where growth rates decreased by 79.2% and 78.6% for O113 and O26, respectively. The growth rate of* Z. bailii* was also influenced when grown in combination with O113 (9.9%) and 026 (11.7%), but clearly this interaction had the greater effect on the bacterial strains. These results are not entirely unexpected since the production of organic acids [18] or ethanol by yeasts in restraining the growth of some microorganisms is not uncommon [19]. However, it was necessary to determine changes in toxicity in mixed cultures in order to investigate the survival adaptation of the bacterial strains in terms of LPS. According to Giotis et al. [20] extended exposure to chemicals in the media might have an effect on growth, which results in the organisms competing with each other for nutrients, leading to poor growth of target organisms.

### 3.2. Influence of Sanitizers on LPS and EPS Toxicity

**Figure 1:**
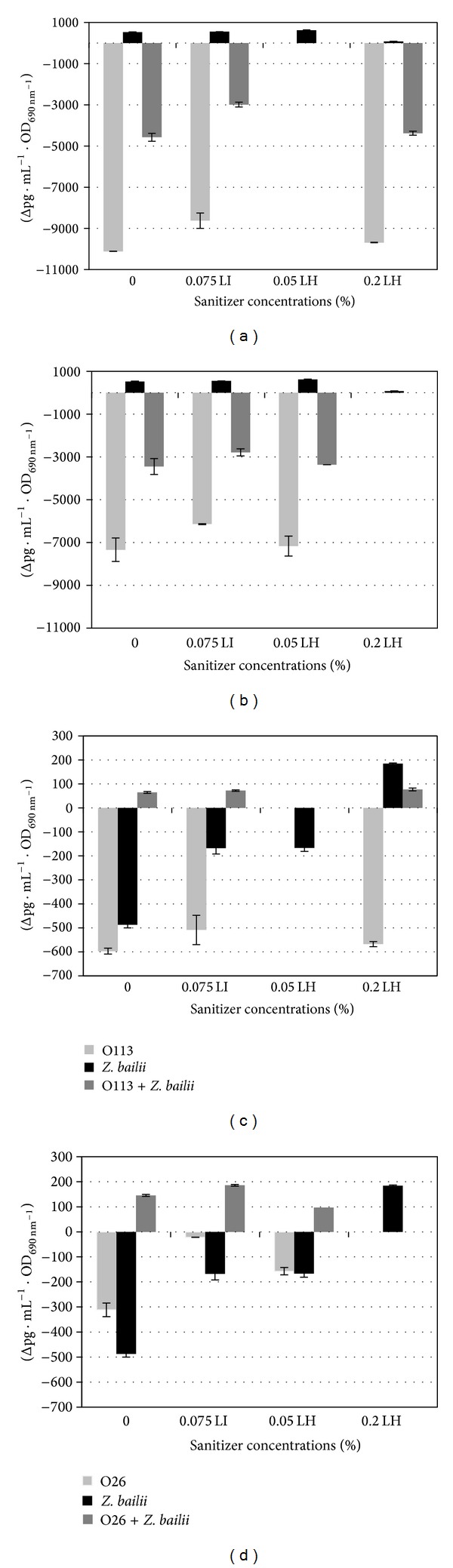
Changes in EPS/LPS toxicity of pure and mixed cultures of* E. coli *and* Z. bailii *following exposure to different concentrations of liquid iodophor and liquid hypochlorite.

For both* E. coli* strains (O113 and O26) growth without sanitizer or in the presence of sanitizers resulted in no increase in toxicity (Figure 1).

When* E. coli O113* was grown without sanitizers (control) a pronounced change in toxicity was observed as a decrease. The same trend was evident in the presence of both sanitizers indicating that the addition of sanitizers to the medium did not influence the toxicity of O113 in pure culture. Growth of* E. coli* O26 also resulted in a decrease in toxicity with the change over time. Exposure to sanitizers still resulted in a decrease in toxicity, in both sanitizers. However, there is a difference in the magnitude of change in the toxicity of O26 in the presence of 0.075% LI (Figure 1(d)). This can be explained by the different modes of action displayed by the sanitizers. Liquid hypochlorite is a highly active oxidizing agent and thereby destroys the cellular activity of proteins. However, their penetration is maximal when they are in a unionized state [21]. Liquid iodophor rapidly penetrates into microorganisms and attacks proteins, nucleotides, and fatty acids; by attacking fatty acids, it already interferes with the lipid A structure measured by LAL method.* Zygosaccharomyces bailii* grown in pure cultures revealed higher toxicity levels when compared to both* E. coli* strains using LAL assay. Limulus amoebocyte lysate interacts with the lipid component of Gram-negative LPS. Although the toxicity values are considerably lower than that detected for the Gram-negative bacteria, this is the first evidence of toxicity detected from eukaryal EPS using the LAL assay. It is tempting to speculate on the similarities that might exist between the targeted sections of the LPS and EPS as has previously been described for Gram-positive EPS [22]. Notably the communal growth of the* E. coli* strains and* Z. bailii* produced different toxicity profiles when using the IL-6 method. In both cases where* E. coli* O113 (Figure 1(c)) or O26 (Figure 1(d)) were cultured in the presence of* Z. bailii* the toxicity of the mixture increased. This occurrence was evident in the presence of both sanitizers indicating that it may be attributed to communal growth. The largest increase in toxicity was brought about by the communal growth of* E. coli* O26 and* Z. bailii* in the presence of sublethal concentration of sanitizer LI. Limulus amoebocyte lysate method on the other hand detected a decrease in toxicity for both O113 (Figure 1(a)) and O26 (Figure 1(b)). The increase in toxicity in communal growth could be a result of cultivation conditions. Communal growth might have influenced the liberation of 3-hydroxy fatty acids resulting in a significant change in saccharide composition of EPS, which affects immune stimulation [14, 23].

Interleukin-6 compared to the LAL assay may be influenced by several parameters which have to be standardized. For example, the whole procedure of LAL assay takes 1 to 3 h, while it takes 4 to 5 h for the IL-6 rendering it time consuming and laborious. Differences between the LAL and IL-6 methods may also depend on the samples used, for example, communal growth samples, showing low values of LAL assay in spite of high toxicity values in IL-6 because the porcine IL-6 is able to detect other pyrogens (e.g., EPS in yeast). In contrast the LAL assay is highly sensitive to endotoxin activity [24].

## 4. Conclusions

In light of these results care should be taken in the food industry where contamination with sanitizers is a risk factor. Incorrect dosage can increase growth of spoiler/pathogenic yeasts. To eliminate such contaminants, food processing equipment needs to be thoroughly rinsed. Factors responsible for the different responses of* E. coli *and* Z. bailii* to the presence of sanitizers, including maximum specific growth rates and toxicity profiles, should be further investigated.

*Escherichia coli *and* Z. bailii* mixed cultures showed increase in toxicity using IL-6 analysis indicating that the use of LAL for the detection of toxicity in a mixed microorganism environment overlooks the contribution made by spoilage yeast. The importance of using the appropriate method for toxicity testing in mixed microbe environments was clearly demonstrated. Although the LAL assay is regarded as sensitive, reproducible, and simplistic, it did not give a representative account of yeast EPS toxicity in mixed cultures, as is relevant to the food processing environment.

## Figures and Tables

**Table 1:** Growth parameters of pure and mixed cultures of *E. coli*  O113, *E. coli*  O26, and *Z. bailii* Y-1535 grown in YM medium in the presence of the following sanitizer concentrations: 0%, 0.05% LH, 0.2% LH, and 0.075% LI.

			*E. coli* O113	*E. coli* O26	*Z. bailii* Y-1535	O113 (ZB + O113)	ZB (ZB + O113)	O26 (ZB + O26)	ZB (ZB + O26)
		*μ*_max⁡_ (h^−1^)	0.496	0.636	0.333	0.103	0.300	0.36	0.294
		*r* ^2^	0.99	0.99	0.99	0.99	0.99	0.99	0.96

LI	0.075%	*μ*_max⁡_ (h^−1^)	0.488	0.629	0.306	0.073	0.259	0.099	0.249
		*r* ^2^	0.99	0.98	0.98	0.99	0.99	0.99	0.96

LH	0.2%	*μ*_max⁡_ (h^−1^)	0.176	—	0.381	0.23	0.352		
		*r* ^2^	0.99	—	0.98	0.76	0.99		

LH	0.05%	*μ*_max⁡_ (h^−1^)	—	0.589	0.259			0.0032	0.355
		*r* ^2^	—	0.99	0.99			0.71	0.98

ZB: *Z. bailii* Y-1535.

(ZB + O113): *Z*. *bailii* cultivated together with *E. coli* O113.

(ZB + O26): *Z. bailii* cultivated together with *E. coli* O26.

*μ*_max⁡_: maximum specific growth rate.
